# Dementia care in developing countries: The road ahead

**Published:** 2009-01

**Authors:** K. S. Shaji

**Affiliations:** Associate Professor of Psychiatry, Medical College, Thrissur - 680596, Kerala, India

Demographic aging is a global phenomenon with differential impact on world regions. It began early in developed countries and progressed over longer period of time. They had more time to develop services. Regions like Asia, Latin America and Africa are now witnessing rapid demographic aging. The developing countries in this region will have comparatively little time to develop services. It is this unprecedented pace of demographic aging which makes it such a huge public health challenge for the region.

Though the biological phenomenon of aging is universal, the daily life of an older person varies considerably according to social, economic, and cultural contexts. Aging and health has many socio-cultural determinants. Social status and available social support for older people vary in different cultures. In many developing countries, older persons are accorded great respect, both within the families and in society. But the traditionally strong social support systems seem to be under strain due to the rapid social restructuring and economic changes sweeping the region.

## DEMENTIA: AN EMERGING PUBLIC HEALTH CHALLENGE

Despite mortality due to communicable diseases, poverty and human conflicts, dementia incidence is destined to increase in the developing world in tandem with the ageing population.[1] According to the Alzheimer’s disease International (ADI) Delphi consensus study, by 2040, 71% of all people with dementia will be living in developing countries. It is estimated that there are about 1.5 million people with dementia in India (compared with 2.9 million in the USA).This number is likely to increase by 300% in the next four decades.[2] This estimate is based on the premise that the Indian incidence rates are relatively low and will remain stable over time. The relatively high prevalence of smoking and the high and rising prevalence of type 2 diabetes are matters of concern in India. These coupled with anticipated life style changes could affect the incidence rates and lead to a higher than expected prevalence in the near future.[3]

The levels of caregiver strain, including that contributed by behavioural disturbances and stress are as high as in developed countries despite extended family networks and home care. Dependency, moreover, is strongly linked to poverty, and imposes additional economic strain on families.[4] People with dementia are cared for at home, by their families. Most families cannot afford institutional care, which in any case is, unavailable in most parts of the developing world.

## HOME BASED CARE

In most developing countries, older people, whether or not widowed, typically live with their families in multi-generational households. This is a good social situation as there are more people in the household to share the responsibility of care. However, populations are becoming increasingly mobile in low income countries. More women are taking up employment out side their homes to supplement the family income. Consequently the caring responsibilities at home will have to be shared by all. It is no more the exclusive responsibility of the traditional housewife.

Many developed countries have comprehensive health and social care systems, but even in such settings families do play a vital caring role in looking after older adults. Interestingly in developing countries, the reliability and universality of the family care system is often overestimated.[5] Assisted living facilities or institutions which provide long-term care are few in low income countries. This puts enormous pressure on the families and caregivers as home based care is the only available option to most people.

## PUBLIC HEALTH OF CAREGIVING

Caregiving itself can be viewed from a public health perspective.[6] If the caregiver remains healthy, then qualify of life of the care recipient will be better. Conversely, a failure in the health of the caregiver may mean collapse of the fragile support systems. In many respects the physical and mental health of the caregiver is at the core of successful caregiving. If the health of the caregiver is better, then he or she is more likely to sustain the caregiving role.

The heaviest load of informal caring responsibilities falls on partners or children. Women are more likely to be carers than men. The provision of intensive informal care to frail older people can have profound consequences for the carer, particularly when the older person is cognitively impaired. Intensive caring can have adverse effects on the psychological health of caregivers. There is consistent evidence that they are more at risk for development of mental health problems, particularly stress and depression, than other adults of the same age.

The challenge for public health systems is to understand more about those caregivers who are particularly vulnerable and why. We need to then design and implement evidence based interventions to address their identified needs. Researchers need to be at the forefront in uncovering possible risk factors associated with the endless types of caregiving situations. From a public health perspective, it is critically important to identify the hazards of caregiving as well as to develop potential improvements and solutions.[6]

## RESEARCH AND SERVICE DEVELOPMENT

The networking of Indian dementia researchers began in 1998 at Cochin with the formation of 10/66 Dementia Research Group. Seven groups of researchers from six centres in India took part in two pilot studies.[4,7] A case finding method was developed for identification of cases in the community.[8] The Indian network of 10/66 Dementia Research Group developed a community based intervention programme. The intervention includes provision of information and education about dementia, sustained carer support and guidance in managing symptoms of dementia. Intervention trials from India and Russia have reported highly promising results.[9,10] However, development of large scale psychogeriatric or dementia care services is not feasible in the developing world.[11] We need to integrate dementia care with general health care by adding a dementia care component to the existing services. We can equip the outreach services to support home based care of people with dementia. Integration with general health care or geriatric care will help to scale up dementia care services in the region.

## LESSONS FROM PALLIATIVE CARE

A few a few years back, the Pain and Palliative Care Society developed a network of palliative care clinics across Kerala, India. Trained volunteers from the community assist in providing care. Family members were empowered to ensure continuity of treatment.[12] Over a period time this has transformed itself into a mass movement in northern districts of Kerala. This has now become a reliable resource for community care and provides strong support for home based care.

An off shoot of this community care movement was “Pariraksha” a joint venture by the District Panchayat (local government) in Malappuram in Kerala and groups engaged in palliative care. “Pariraksha” means “protective care”. It is a broader initiative aimed at meeting the care needs of all community resident disabled individuals. They offer support and guidance to all chronically and incurably ill patients in the district. They have established home care programs with community participation. Volunteers from the local community are trained to identify problems of the chronically ill in their area and to intervene effectively, with active support from a network of trained professionals. Management and coordination of the program is also by community volunteers. Interestingly, their clients include a large number of functionally impaired older adults, many with dementia.

We could make this even more effective with sustained inputs from various specialists involved in management of chronic diseases. “Pariraksha” viable model for community care of older people with functional impairment, especially those with conditions like dementia. The 10/66 Dementia research group at Thrissur is working with local community care initiatives to integrate dementia care into their services. Broadening the scope of palliative care services to include dementia care, would be a welcome step particularly in the developing world. But those who deliver caregiver interventions should receive training. The interventions provided should take into consideration the specific needs of dementia care while remaining adhered to the general principles of palliative care. The intervention module developed by the Indian network of 10/66 Dementia Research Group meets this criterion. Details are available at http://www.alz.co.uk/1066.

## NEED FOR SPECIALISED SERVICES

We should not neglect the development of specialised dementia care services in developing countries. We need to facilitate the provision of clinic based services in general hospitals and medical colleges. Dementia clinics or memory clinics are examples of such services. Some centres can focus on geriatric psychiatry and dementia care. Development and networking of centres with special interest in dementia is also important. Such centres will help clinicians to acquire skills, knowledge and above all, hands on experience in specialised dementia care. Expertise of those clinicians can be used for training. It is essential to develop geriatric psychiatry as a subspecialty. A beginning has to be made in this direction by setting up of departments of geriatric psychiatry or division of geriatric psychiatry in major hospitals in developing countries.

## FUTURE DIRECTIONS

Attention needs to be directed towards the development of age-appropriate long-term care policy. There has to be mechanisms for ensuring the social protection of older persons. Dementia is an important cause of dependency among older people in the developing world.[13] We must convince the governments and policy makers the urgent need to face the public health challenges posed by dementia. They need to be informed about the possible responses to handle this challenge. The priorities should be made clear. The first priority is to identify and support home based care of people with dementia. Dementia care has to be integrated with general health care. Outreach services should develop the capacity to deliver caregiver interventions.

Community based services, based on principles of palliative care can address the needs of people affected by a wide variety of disabling and incurable conditions. It should lead to the development of locally sustainable home care programs. A network of nurses and doctors with expertise in palliative care can supervise and support such initiatives. Dementia care can be delivered as part of such initiatives. Continuing care units for people with dementia might consider themselves as specialist palliative care units.[14] Through outreach and liaison, they could foster broad palliative care for people with dementia in the community. Their expertise should be of great help in the management of behavioural symptoms.[15] This model might work well in most settings if suitably adapted to meet the local aspirations.

The second priority is the development of specialised dementia care services in general hospitals. We need more clinicians with expertise in dementia care in developing countries. We also need more centres with special interest in dementia care. This will provide more opportunities for training in dementia care. Government and professional organisations can take lead in this regard. The third priority is to encourage good quality research. We need to develop an evidence base for the region. Research shall guide service development. We did make some progress in the last few years.[16] But, there is a long way to go and there is no time to lose.
